# Ketone bodies and cancer risk in the general population: The prevention of renal and vascular end‐stage disease (PREVEND) study

**DOI:** 10.1111/eci.70100

**Published:** 2025-07-23

**Authors:** Li Luo, Martine G. E. Knol, Lyanne M. Kieneker, Bert van der Vegt, Stephan J. L. Bakker, Eke G. Gruppen, Rudolf A. de Boer, Joseph Pierre Aboumsallem, Margery A. Connelly, Robin P. F. Dullaart, Ron T. Gansevoort

**Affiliations:** ^1^ Department of Nephrology University Medical Center Groningen, University of Groningen Groningen The Netherlands; ^2^ Department of Pathology University Medical Center Groningen, University of Groningen Groningen The Netherlands; ^3^ Department of Cardiology Thorax Center, Erasmus MC, Cardiovascular Institute Rotterdam The Netherlands; ^4^ Labcorp Morrisville North Carolina USA; ^5^ Department of Endocrinology University Medical Center Groningen, University of Groningen Groningen The Netherlands

**Keywords:** cancer, ketone bodies, malignancy, population‐based study

## 1 INTRODUCTION

Ketone bodies (KBs) comprise acetoacetate (AcAc), acetone and β‐hydroxybutyrate (β‐OHB). In the state of limited glucose supply, KBs are produced from fatty acids in the liver and transported to the extrahepatic cells as an alternative source of energy. Research interest in the association between KBs and cancer is growing, largely because a ketogenic diet has been reported to have the potential to delay cancer development by starving proliferating tumour cells, which highly depend on aerobic glycolysis and thus cannot use KBs that are produced during fasting as an alternative source of energy (i.e., the Warburg effect).^1^

Conversely, metabolomic studies have shown that higher KBs, as a sign of metabolic dysregulation, are associated with cancer development and progression.^2^, ^3^ One possible reason for these conflicting findings between KB levels and cancer incidence may be the lack of adjustment for important confounders.^4^ Another possible explanation can be differences in study populations of cancer patients versus the general population, as the effects of KBs on cancer may function through unique pathways subject to a specific pathophysiological context. For example, the effect of KBs in sensitising cancer cells to radiotherapy only applies to cancer patients, not to healthy individuals. Of note, available cohort studies on the association between KBs and cancer in the general population are scarce yet important, as KBs have been suggested to be promising markers to screen for cancer.^5^

Taking these considerations together, we aimed to examine whether and how KBs are associated with cancer incidence in a prospective population‐based cohort study with extensive adjustment for confounders.

## 2 MATERIALS AND METHODS

For this study, we analysed data from 6079 subjects in the Prevention of Renal and Vascular End‐stage Disease (PREVEND) study, a prospective, population‐based cohort.^6^ Total KBs (AcAc, β‐OHB and acetone) were measured by the plasma‐based NMR method. The primary outcome was the incidence of overall cancer. Secondary outcomes were the incidence of urinary tract, lung and colorectal cancer. Cox regression models were performed to calculate hazard ratios (HRs, 95% CIs), crude and adjusted. Details of the methods are provided in the (Tables S1 and S2, Figure S1).

## 3 RESULTS

### 3.1 Baseline characteristics

Baseline characteristics of the 6079 subjects included in this study are presented in Table 1. Their mean age was 53.9 ± 12.1 years, 50.4% were female, and these subjects were predominantly white. The median level of KBs was 178.8 (IQR, 141.2–244.8) μmol/L. At baseline, subjects in the highest tertile of KBs were more likely to be older, to have a higher level of fasting glucose, to have type 2 diabetes, to exhibit worse lipid profiles and to have a higher level of hs‐CRP when compared to those in the lowest tertile of KBs.

**TABLE 1:** Baseline characteristics of the 6079 PREVEND subjects overall and according to tertiles of ketone bodies.

	Total	Tertiles of ketone bodies (μmol/L)			*p* for trend
		<152.6	152.6–216.1	≥216.1	
Participants, *n*	6079	2026	2026	2027	
Age, y	53.9 ± 12.1	51.3 ± 11.3	54.4 ± 12.0	56.1 ± 12.4	<.001
Female sex, %	3064 (50.4%)	1065 (52.6%)	957 (47.2%)	1042 (51.4%)	.002
Whites, %	5789 (96.0%)	1916 (95.4%)	1951 (96.9%)	1922 (95.7%)	.036
BMI, kg/m^2^	26.8 ± 4.4	26.1 ± 4.0	27.1 ± 4.3	27.1 ± 4.7	<.001
SBP, mmHg	126.4 (18.8)	123.5 (17.5)	127.1 (18.4)	128.5 (20.2)	<.001
Smoking status, current, %	1685 (28.1)	568 (28.5)	546 (27.2)	571 (28.5)	.572
Alcohol consumption					
None, %	1546 (25.7)	532 (26.6)	490 (24.4)	524 (26.1)	<.001
Occasional, %	2912 (47.9)	1007 (49.7)	1016 (50.1)	889 (43.9)	
Moderate, %	1323 (21.8)	415 (20.5)	425 (21.0)	483 (23.8)	
Heavy, %	239 (3.9)	49 (2.4)	80 (3.9)	110 (5.4)	
Education, high, %	1802 (29.6)	630 (31.1)	593 (29.3)	579 (28.6)	.19
Hypertension, %	2092 (34.5)	547 (27.0)	740 (36.6)	805 (39.8)	<.001
Type 2 diabetes, %	384 (6.4)	49 (2.5)	119 (6.0)	216 (10.8)	<.001
Cancer history (any type), %	254 (4.2)	66 (3.3)	79 (3.9)	109 (5.4)	.003
Ketone bodies, umol/L	178.8 (141.2, 244.8)	128.6 (112.8, 141.2)	178.8 (164.3, 196.0)	291.4 (244.8, 383.8)	<.001
β‐OHB, μmol/L	122.3 (94.9, 167.0)	86.7 (74.8, 97.8)	122.3 (109.6, 135.2)	197.9 (166.3, 258.5)	<.001
AcAc, μmol/L	38.4 (26.2, 56.3)	25.1 (18.4, 31.8)	37.4 (28.9, 46.5)	67.4 (51.5, 90.7)	<.001
Acetone, μmol/L	19.6 (12.9, 28.5)	13.5 (8.9, 18.8)	19.0 (13.7, 25.3)	30.2 (22.1, 40.4)	<.001
Glucose, mmol/L	5.1 ± 1.2	4.9 ± .8	5.1 ± 1.0	5.2 ± 1.5	<.001
C‐Peptide, pmol/L	746.0 (579.0, 990.0)	689.0 (553.0, 887.0)	786.0 (611.0, 1043.5)	773.0 (585.0, 1042.0)	<.001
HDL cholesterol, mg/dL	48.3 ± 11.8	49.4 ± 11.5	47.4 ± 11.7	48.1 ± 12.1	<.001
Triglycerides, mg/dL	119.9 ± 77.2	106.2 ± 61.0	125.5 ± 69.9	128.1 ± 94.7	<.001
hs‐CRP, mg/L	1.4 (.7–3.1)	1.0 (.5–2.0)	1.6 (.7–3.2)	1.9 (.8–4.1)	<.001
Plasma pre‐albumin, g/L	.3 ± .1	.3 ± .0	.3 ± .0	.3 ± .1	.027
Plasma albumin, g/L	43.8 ± 4.9	43.9 ± 2.7	43.8 ± 5.3	43.8 ± 5.9	.651
eGFR, mL/min/1.73m^2^	91.2 ± 17.4	93.8 ± 16.4	91.0 ± 17.3	88.7 ± 18.0	<.001

*Note*: *p* for trend is determined by a *χ*2 test (categorical variables), or by linear regression or a Kruskal–Wallis test (continuous variables).

Abbreviations: AcAc, acetoacetate; BMI, body mass index; hs‐CRP, high sensitivity C‐reactive protein; eGFR, estimated glomerular filtration rate; HDL, high‐density lipoprotein; β‐OHB, β‐hydroxybutyrate; PREVEND, the Prevention of Renal and Vascular End‐stage Disease study; SBP, systolic blood pressure.

### 3.2 Ketone bodies and the risk of overall cancer incidence

During a median follow‐up of 13.4 years, 828 subjects developed cancer. Each doubling of KBs was associated with a 34% (HR, 1.34, 95% CI, 1.20–1.49, model 1) higher risk of overall cancer incidence (Table 2). The magnitude of this effect of KBs was attenuated to 14% (HR, 1.14, 95% CI, 1.02–1.27, model 2) when adjusting for age and sex, and to 13% (HR, 1.13, 95% CI, 1.01–1.26, model 3) when additionally adjusting for coexisting conditions. Adjusting for metabolic profiles (model 4a) did not substantially change the association when compared to model 3. However, the association between doubling of KBs and overall cancer incidence became nonsignificant when additionally adjusting for hs‐CRP (HR, 1.09, 95% CI, .97–1.23, mode 4b). These results remained consistent in the sensitivity analysis which specifically examined β‐OHB as the study exposure (Table S3). We did not find effect modification by age, sex, BMI and type 2 diabetes in the association of KBs and the incidence of overall cancer (Figure S2, all *p*_interaction_ > .05). Results of the mediation analysis showed that hs‐CRP partly mediated the association between KBs and overall cancer incidence (proportion mediated, 21.0%, 95% CI, 8.1%–73.0%, Table S4).

**TABLE 2:** Associations of ketone bodies concentration with the overall incidence of cancer and with the incidence of the most common site‐specific cancers during follow‐up.

	Ketone bodies per doubling, HR (95% CI)	*p* Value
Overall cancer		
No. of events/subjects	828/5825	
Model 1	1.34 (1.20–1.49)	<.001
Model 2	1.14 (1.02–1.27)	.023
Model 3	1.13 (1.01–1.26)	.040
Model 4a	1.15 (1.02–1.29)	.019
Model 4b	1.10 (.98–1.24)	.119
Urinary tract cancer		
No. of events/subjects	108/6049	
Model 1	1.43 (1.07–1.91)	.015
Model 2	1.13 (.83–1.54)	.454
Model 3	1.07 (.78–1.47)	.657
Model 4a	1.09 (.78–1.52)	.607
Model 4b	1.00 (.72–1.40)	.993
Lung cancer		
No. of events/subjects	150/6065	
Model 1	1.33 (1.04–1.71)	.023
Model 2	1.05 (.81–1.37)	.706
Model 3	1.02 (.78–1.33)	.895
Model 4a	.98 (.74–1.28)	.861
Model 4b	.93 (.70–1.24)	.611
Colorectal cancer		
No. of events/subjects	118/6055	
Model 1	1.42 (1.08–1.88)	.013
Model 2	1.15 (.86–1.55)	.340
Model 3	1.13 (.84–1.52)	.423
Model 4a	1.13 (.83–1.54)	.446
Model 4b	1.12 (.82–1.54)	.467

*Note*: HRs, 95% Cis and *p* values were derived from Cox proportional hazards regression models. Model 1: crude; Model 2: adjusted for age and sex; Model 3: model 2 + adjusted for BMI, alcohol, eGFR and type 2 diabetes; Model 4a: model 3 + adjusted for fasting glucose, C‐Peptide, triglycerides and HDL cholesterol; Model 4b: model 3 + adjusted for hs‐CRP.

Abbreviations: BMI, body mass index; CI, confidence interval; eGFR, estimated glomerular filtration rate; HDL, high‐density lipoprotein; HR, hazard ratio; hs‐CRP, high sensitivity C‐reactive protein.

### 3.3 Ketone bodies and the risk of site‐specific cancer incidence

Each doubling of KBs was associated with a higher risk of incident urinary tract (HR, 1.43, 95% CI, 1.07–1.91), lung (HR, 1.33, 95% CI, 1.04–1.71) and colorectal cancer (HR, 1.42, 95% CI, 1.08–1.88, Table 2). Most of these associations were attenuated to null after adjustment for age and sex, and lost even more strength after multivariable adjustment for confounders (HR for incident urinary tract cancer, 1.07, 95% CI, .78–1.47; HR for incident lung cancer, 1.02, 95% CI, .78–1.33; HR for colorectal cancer, 1.13, 95% CI, .84–1.52, all model 3).

## 4 DISCUSSION

While our study found no association between KBs and cancer development, several studies suggested potential protective effects of KBs on cancer. Notably, most of these studies suggesting the antitumour effects of increasing KBs were conducted in the context of a ketogenic diet designed specifically for cancer patients.^7^, ^8^ One reason why the direction of the association between KBs and cancer is different between our findings and these studies may be that the beneficial effects of KBs on cancer are unique for patients with prevalent cancer (e.g., discriminately intensify the effects of anticancer therapy for tumour cells). An alternative explanation is that therapeutically induced KBs and chronically, spontaneously elevated KBs may have different effects on cancer development. Several authors have argued that therapeutically induced KBs can positively influence body composition and enhance the anticancer effects from radio‐ and chemotherapy,^9^ while chronically elevated KBs may be related to pro‐tumour modulation as an onco‐metabolite or act as a confounding factor, as described further below.^8^ Of note, marked elevations in circulating KB concentrations among the healthy general population are usually observed only in certain situations, including severe exercise, extended fasting or maintaining a ketogenic diet. Given that >99% of subjects of our study population had β‐OHB concentrations within the normal range (.5 mM), and that the use of ketogenic diets was not widely introduced or recommended to the general population in the era when PREVEND subjects were included, the associations between KBs and cancer risk documented in our study are highly likely to reflect the associations for chronically, spontaneously elevated KBs. Importantly, there is accumulating evidence suggesting that chronically elevated KBs are involved in subclinical metabolic alterations.^10^, ^11^ Additionally, this also indicates that our study cannot rule out the potential benefits of KBs induced by ketogenic diet on slowing cancer progression.

To date, a few metabolomic profiling studies have also shown a positive association between KBs and cancer, which we did not find in the current study.^5^, ^12^ One of the possible explanations for the discrepant findings can be the lack of controlling for important confounders (e.g., type 2 diabetes and inflammation) in these metabolomics studies. This is crucial because it may not be the direct effects of increasing KBs but the underlying factor driving the increase in KBs that has pro‐tumour effects, as suggested in our data by the attenuation in the association between KBs and cancer incidence after adjusting for confounders.

Several underlying stimuli of increasing KBs may confound the positive association between chronically elevated KBs and cancer. First, a higher level of KBs may capture subjects with older age in the general population, as KBs can re‐balance the decreased mitochondrial pyruvate dehydrogenase complex activity related to aging.^13^ As indicated by the attenuation of the association between KBs and cancer incidence after adjusting for age in our data, it is likely that KBs per se do not predict higher cancer risk, but that the older subjects captured by higher KBs are intrinsically at a higher risk of developing cancer. Second, although there is no specific indication in our data, metabolic dysregulation itself may play a role in the observed association between KBs and cancer. In other words, the increase in KBs may indicate the process of maintaining energy homeostasis in response to metabolic dysregulation.^14^ Third, the increase in systemic inflammation that may occur in parallel with increasing KBs is another crucial consideration. Since inflammation is a well‐established risk factor driving carcinogenesis,^15^ the higher cancer risk associated with increasing KBs may be at least partly explained by the concurrent activation of inflammation. Importantly, while our analyses cannot definitively establish whether inflammation, characterised by hs‐CRP in this study, serves as a mediator or a confounder, our findings suggest that if hs‐CRP acts as a mediator, it may account for approximately 21% of the association between ketone bodies and overall cancer incidence.

Our study had several strengths. To our knowledge, this is the first prospective cohort study to investigate the association of KBs with cancer incidence in the general population. Another strength is the well‐phenotyped cohort, which enables us to explore the effects of several clinically important confounders on the association between KBs and cancer. Additionally, KBs concentration can be measured by analysis of plasma, urine and breath, but the plasma‐based NMR method that we used to measure KBs concentration in this study possesses higher accuracy.^16^ Lastly, data on cancer incidence were verified via record linkage with Palga, the Dutch nationwide pathology databank, which has complete national coverage.^17^ Limitations include that the plasma samples used for measuring KBs were obtained after overnight fasting, meaning KBs concentrations could be slightly higher in the nonfasting state. However, such higher KBs concentrations are expected to consistently inflate the magnitude of the observed association among participants, whereas we found a null association, making our findings potentially more robust.

In conclusion, a higher level of KBs is associated with a higher risk of incident overall, urinary tract, lung and colorectal cancer in the general population, but these associations are nullified after adjustment for clinically important confounders.

## AUTHOR CONTRIBUTIONS

All authors conceived and designed the study. L.M.K., B.v.d.V., E.G.G., S.J.L.B., R.A.d.B., M.A.C., R. P. F. D. and R.T.G. contributed to data acquisition. L.L. conducted data analysis. All authors contributed to the interpretation of the data. L.L., M.G.E.K., L.M.K. and R.T.G. drafted the manuscript. All authors revised the article. L.M.K. and R.T.G. supervised the work. All authors approved the final version of the manuscript.

## FUNDING INFORMATION

The PREVEND study is supported by several grants from the Dutch Kidney Foundation (E.033) and the Dutch Heart Foundation (2001.005), the Dutch Government, the US National Institutes of Health and the University Medical Center Groningen, the Netherlands. L.L. is supported by a scholarship from the China Scholarship Council (CSC number: 202008440376). Dr. de Boer is supported by the European Research Council (ERC CoG 818715).

## CONFLICT OF INTEREST STATEMENT

M.A.C. is an employee of and holds stock in Labcorp. Other authors declare not to have conflicts of interest for the present work.

## Supporting information


Appendix S1.

## Data Availability

The data contain patient‐related information and cannot be shared publicly as per European GDPR regulations. The data can be accessed through collaborative research applications addressed to the principal investigator Ron T. Gansevoort (r.t.gansevoort@umcg.nl), and subjected to data sharing agreements that fulfil institutional and national regulations.

## References

[1] Feng S , Wang H , Liu J , Aa J , Zhou F , Wang G . Multi‐dimensional roles of ketone bodies in cancer biology: opportunities for cancer therapy. Pharmacol Res. 2019;150:104500. doi:10.1016/j.phrs.2019.104500 31629092

[2] Rocha CM , Carrola J , Barros AS , et al. Metabolic signatures of lung cancer in biofluids: NMR‐based metabonomics of blood plasma. J Proteome Res. 2011;10(9):4314‐4324. doi:10.1021/pr200550p 21744875

[3] Huang J , Mondul AM , Weinstein SJ , et al. Prospective serum metabolomic profiling of lethal prostate cancer. Int J Cancer. 2019;145(12):3231‐3243. doi:10.1002/ijc.32218 30779128 PMC6698432

[4] Shahid RK , Ahmed S , Le D , Yadav S . Diabetes and cancer: risk, challenges, management and outcomes. Cancers (Basel). 2021;13(22):5735. doi:10.3390/cancers13225735 34830886 PMC8616213

[5] Turkoglu O , Zeb A , Graham S , et al. Metabolomics of biomarker discovery in ovarian cancer: a systematic review of the current literature. Metabolomics. 2016;12(4):60. doi:10.1007/s11306-016-0990-0 28819352 PMC5557039

[6] Lambers Heerspink HJ , Brantsma AH , De Zeeuw D , Bakker SJL , De Jong PE , Gansevoort RT . Albuminuria assessed from first‐morning‐void urine samples versus 24‐hour urine collections as a predictor of cardiovascular morbidity and mortality. Am J Epidemiol. 2008;168(8):897‐905. doi:10.1093/aje/kwn209 18775924

[7] Santos JG , Da Cruz WMS , Schönthal AH , et al. Efficacy of a ketogenic diet with concomitant intranasal perillyl alcohol as a novel strategy for the therapy of recurrent glioblastoma. Oncol Lett. 2018;15(1):1263‐1270. doi:10.3892/ol.2017.7362 29391903 PMC5769394

[8] Weber DD , Aminzadeh‐Gohari S , Tulipan J , Catalano L , Feichtinger RG , Kofler B . Ketogenic diet in the treatment of cancer – where do we stand? Mol Metab. 2020;33:102‐121. doi:10.1016/j.molmet.2019.06.026 31399389 PMC7056920

[9] Klement RJ . Beneficial effects of ketogenic diets for cancer patients: a realist review with focus on evidence and confirmation. Med Oncol. 2017;34(8):132. doi:10.1007/s12032-017-0991-5 28653283

[10] Post A , Garcia E , van den Berg EH , et al. Nonalcoholic fatty liver disease, circulating ketone bodies and all‐cause mortality in a general population‐based cohort. Eur J Clin Investig. 2021;51(12):1‐12. doi:10.1111/eci.13627 PMC9285047 34120339

[11] Niezen S , Connelly MA , Hirsch C , et al. Elevated plasma levels of ketone bodies are associated with all‐cause mortality and incidence of heart failure in older adults: the CHS. J Am Heart Assoc. 2023;12(17):1‐11. doi:10.1161/JAHA.123.029960 PMC10547348 37609928

[12] Sanchez‐Espiridion B , Liang D , Ajani JA , et al. Identification of serum markers of esophageal adenocarcinoma by global and targeted metabolic profiling. Clin Gastroenterol Hepatol. 2015;13(10):1730‐1737.e9. doi:10.1016/j.cgh.2015.05.023 25998788 PMC4596233

[13] Veech RL , Bradshaw PC , Clarke K , Curtis W , Pawlosky R , King MT . Ketone bodies mimic the life span extending properties of caloric restriction. IUBMB Life. 2017;69(5):305‐314. doi:10.1002/iub.1627 28371201

[14] van der Vaart A , Knol MGE , de Borst MH , et al. The paradoxical role of circulating ketone bodies in glycemic control of individuals with type 2 diabetes: high risk, high reward? Biomolecules. 2022;12(9):1318. doi:10.3390/biom12091318 36139157 PMC9496560

[15] Guo YZ , Pan L , Du CJ , Ren DQ , Xie XM . Association between C‐reactive protein and risk of cancer: a meta‐analysis of prospective cohort studies. Asian Pac J Cancer Prev. 2013;14(1):243‐248. doi:10.7314/APJCP.2013.14.1.243 23534731

[16] Shemesh E , Chevli PA , Islam T , et al. Circulating ketone bodies and cardiovascular outcomes: the MESA study. Eur Heart J. 2023;44(18):1636‐1646. doi:10.1093/eurheartj/ehad087 36881667 PMC10411932

[17] Casparie M , Tiebosch ATMG , Burger G , et al. Pathology databanking and biobanking in the Netherlands, a central role for PALGA, the nationwide histopathology and cytopathology data network and archive. Cell Oncol. 2007;29(1):19‐24. doi:10.1155/2007/971816 17429138 PMC4618410

