# Supplementary material for: Ketone bodies and cancer risk in the general population: The prevention of renal and vascular end‐stage disease (PREVEND) study
Source: Eur J Clin Invest. 2025 Jul 23;55(10):e70100. doi: 10.1111/eci.70100 (PMC12434450; doi:10.1111/eci.70100)
Supplement: Supplementary file 1 — Appendix S1. [file ECI-55-e70100-s001.docx]

**Table S1.** Missing values of the covariates.

| Variables | Proportion of the missing values |
| --- | --- |
| BMI, kg/m^2^ | 0.03% |
| Smoking status, % | 1.25% |
| Alcohol consumption, % | 0.97% |
| Type 2 diabetes, % | 1.86% |
| Glucose, mmol/L | 1.74% |
| C-Peptide, pmol/L | 2.88% |
| hs-CRP, log2 | 5.64% |
| Plasma pre-albumin, g/L | 6.81% |
| Plasma albumin, g/L | 7.12% |

Abbreviations: BMI, body mass index; HDL, high-density lipoprotein; HR, hazard ratio; hs-CRP, high sensitivity C-reactive protein.

**Table S2.** Univariable linear and Cox regression analyses investigating the association of potential confounders with ketone bodies concentration and overall cancer incidence.

|  | ß | P value for ß | Hazard ratio | P value for hazard ratio |
| --- | --- | --- | --- | --- |
| Age, y | 0.01 | **<0.001** | 1.06 | **<0.001** |
| Female sex, % | 0.00 | 0.864 | 0.70 | **<0.001** |
| BMI, kg/m^2^ | 0.01 | **<0.001** | 1.03 | **<0.001** |
| Smoking status, never, % | ref (0.00) |  | ref (1.00) |  |
| Smoking status, former, % | 0.03 | 0.172 | 2.16 | **<0.001** |
| Smoking status, current, % | 0.00 | 0.936 | 1.94 | **<0.001** |
| Alcohol consumption, none, % | ref (0.00) |  | ref (1.00) |  |
| Alcohol consumption, light, % | -0.03 | **0.092** | 0.94 | 0.480 |
| Alcohol consumption, moderate, % | 0.05 | **0.020** | 1.17 | 0.105 |
| Alcohol consumption, heavy, % | 0.19 | **<0.001** | 1.35 | **0.080** |
| Type 2 diabetes, % | 0.33 | **<0.001** | 1.56 | **<0.001** |
| Glucose, mmol/L | 0.07 | **<0.001** | 1.11 | **<0.001** |
| C-Peptide, pmol/L | 0.09 | **<0.001** | 1.49 | **<0.001** |
| HDL cholesterol, mg/dL | 0.00 | **0.024** | 0.99 | **0.020** |
| Triglycerides, mg/dL | 0.00 | **<0.001** | 1.00 | **0.001** |
| hs-CRP, log2 | 0.08 | **<0.001** | 1.18 | **<0.001** |
| Plasma pre-albumin, g/L | -0.25 | 0.102 | 0.44 | 0.260 |
| Plasma albumin, g/L | 0.00 | 0.278 | 0.94 | **<0.001** |
| eGFR, mL/min/1.73m^2^ | -0.01 | **<0.001** | 0.97 | **<0.001** |
| hs-CRP and C-Peptide were log2 transformed for analyses. | | | | |
| ß is the standardized regression coefficient of the corresponding confounder in the model of regressing ketone bodies on the confounder. | | | | |
| ßs and P values for ßs were derived from linear regression models. Bold P values indicate statistical significance (P <0.10). | | | | |
| HR is the exponentiated regression coefficient of the corresponding confounder in the model of regressing overall cancer incidence on the confounder. | | | | |
| HRs and P values for HRs were derived from Cox proportional hazards regression models. | | | |  |
| Abbreviations: BMI, body mass index; eGFR, estimated glomerular filtration rate; HDL, high-density lipoprotein; HR, hazard ratio; hs-CRP, high sensitivity C-reactive protein. | | | | |

**Table S3.** Associations of β-OHB concentration with the overall incidence of cancer and with the incidence of the most common site-specific cancers during follow-up.

|  | **β-OHB per doubling, HR (95% CI)** | **P value** |
| --- | --- | --- |
| **Overall cancer** |  |  |
| No. of events/subjects | 828/5,825 |  |
| model 1 | 1.12 (1.01-1.25) | 0.034 |
| model 2 | 1.02 (0.97-1.07) | 0.458 |
| model 3 | 1.02 (0.97-1.06) | 0.487 |
| model 4a | 1.02 (0.97-1.06) | 0.418 |
| model 4b | 1.01 (0.97-1.05) | 0.611 |
| **Urinary tract cancer** |  |  |
| No. of events/subjects | 108/6,049 |  |
| model 1 | 1.45 (1.10-1.92) | 0.010 |
| model 2 | 1.19 (0.87-1.59) | 0.291 |
| model 3 | 1.11 (0.82-1.51) | 0.502 |
| model 4a | 1.11 (0.81-1.53) | 0.522 |
| model 4b | 1.05 (0.82-1.35) | 0.687 |
| **Lung cancer** |  |  |
| No. of events/subjects | 150/6,065 |  |
| model 1 | 1.29 (1.01-1.64) | 0.043 |
| model 2 | 1.04 (0.87-1.24) | 0.642 |
| model 3 | 1.04 (0.90-1.20) | 0.632 |
| model 4a | 1.03 (0.92-1.15) | 0.597 |
| model 4b | 1.02 (0.92-1.12) | 0.749 |
| **Colorectal cancer** |  |  |
| No. of events/subjects | 118/6,055 |  |
| model 1 | 1.48 (1.13-1.94) | 0.004 |
| model 2 | 1.22 (0.92-1.63) | 0.176 |
| model 3 | 1.20 (0.90-1.61) | 0.216 |
| model 4a | 1.18 (0.87-1.60) | 0.277 |
| model 4b | 1.19 (0.88-1.62) | 0.265 |
| HRs, 95% Cis and P values were derived from Cox proportional hazards regression models. | | |
| Model 1: crude; |  |  |
| Model 2: adjusted for age and sex; | |  |
| Model 3: model 2 + adjusted for BMI, alcohol, eGFR, and type 2 diabetes; | | |
| Model 4a: model 3 + adjusted for fasting glucose, C-Peptide, triglycerides and HDL cholesterol; | | |
| Model 4b: model 3 + adjusted for hs-CRP. | |  |
| Abbreviations: BMI, body mass index; CI, confidence interval; eGFR, estimated glomerular filtration rate; HDL, high-density lipoprotein; HR, hazard ratio; hs-CRP, high sensitivity C-reactive protein. | | |

**Table S4.** Mediation analysis of the association between ketone bodies and overall cancer incidence via hs-CRP.

| Mediator | Overall cancer incidence | | | | | |
| --- | --- | --- | --- | --- | --- | --- |
|  | No. of cancer events/subjects | ACME, 95% CI | ADE, 95% CI | Total effect, 95% CI | Proportion mediated (%, 95% CI) | P value for proportion mediated |
| hs-CRP, mg/L | 1,268/7,836 | 2.46 (1.19, 3.95) | 9.07 (0.81, 18.00) | 11.53 (3.49, 20.37) | 21.0 (8.1, 73.0) | 0.002 |
| Causal mediation analyses were conducted using the mediate() function from the R package. Effects are reported as proportion mediated of the association between ketone bodies (<177.7 versus ≥177.7 μmol/L, cohort-specific median value) and the incidence of overall cancer. The significance of the mediation effect was tested by the quasi-Bayesian Monte Carlo method with 1000 simulations. Estimates are adjusted for age, sex, BMI, smoking, BMI, alcohol, eGFR, and type 2 diabetes. | | | | | | |
| Abbreviations: ACME, average causal mediation effect; ADE, average direct effect; hs-CRP, high sensitivity C-reactive protein. | | | | | |  |

**Figure S1.** Conceptual DAGs illustrating the role of hs-CRP in the association between ketone bodies and overall cancer incidence.


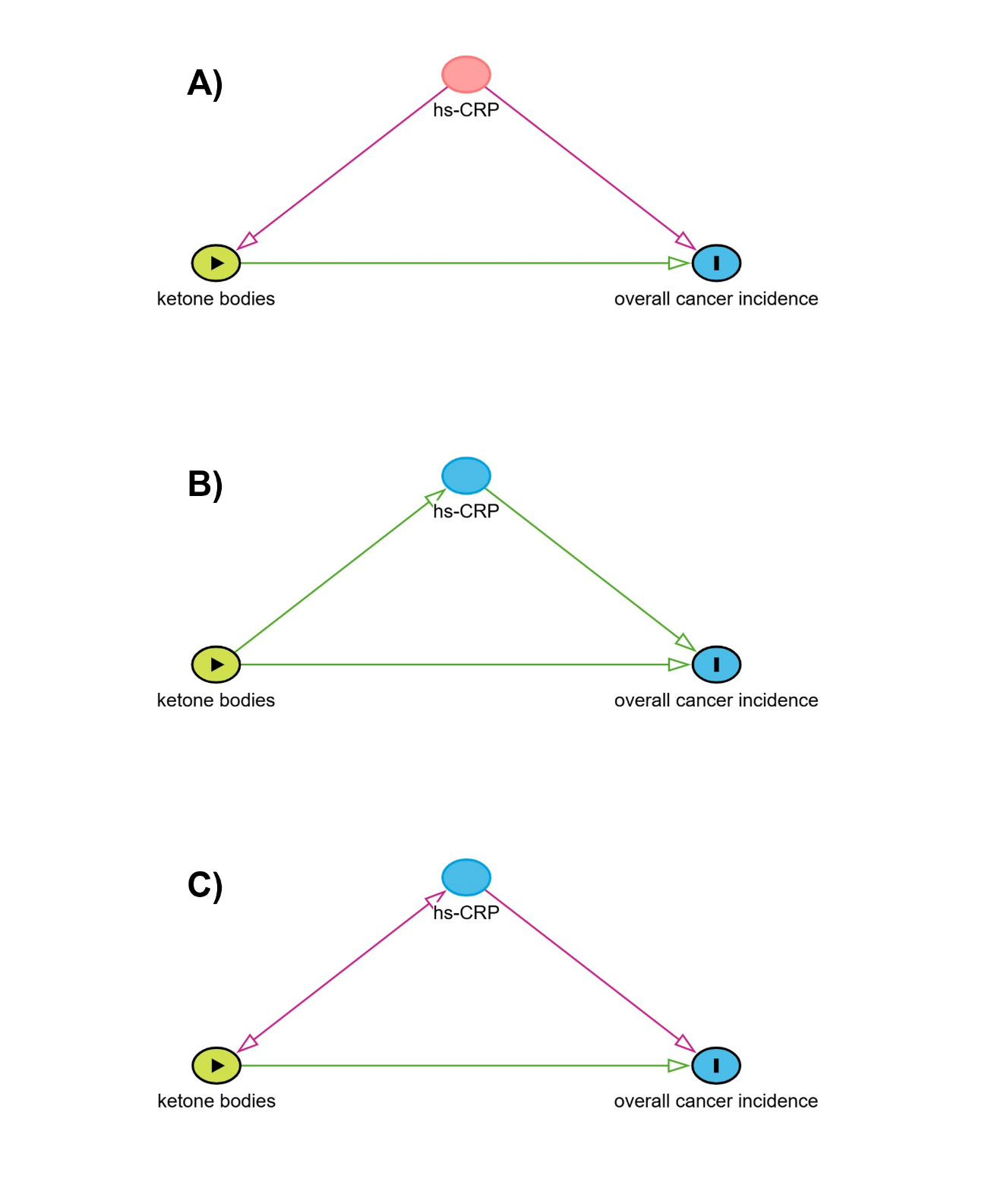


This figure includes three DAGs, which presents three scenarios of hs-CRP in the association between ketone bodies and overall cancer incidence. Panel A: hs-CRP as a confounder; Panel B: hs-CRP as a mediator; Panel C: hs-CRP as both a confounder and a mediator.

Abbreviations: DAGs, directed acyclic graphs; hs-CRP, high sensitivity C-reactive protein.

**Figure S2.** Subgroup analyses investigating effect modification of the association of ketone bodies concentration with cancer incidence by age, sex, BMI and diabetes.


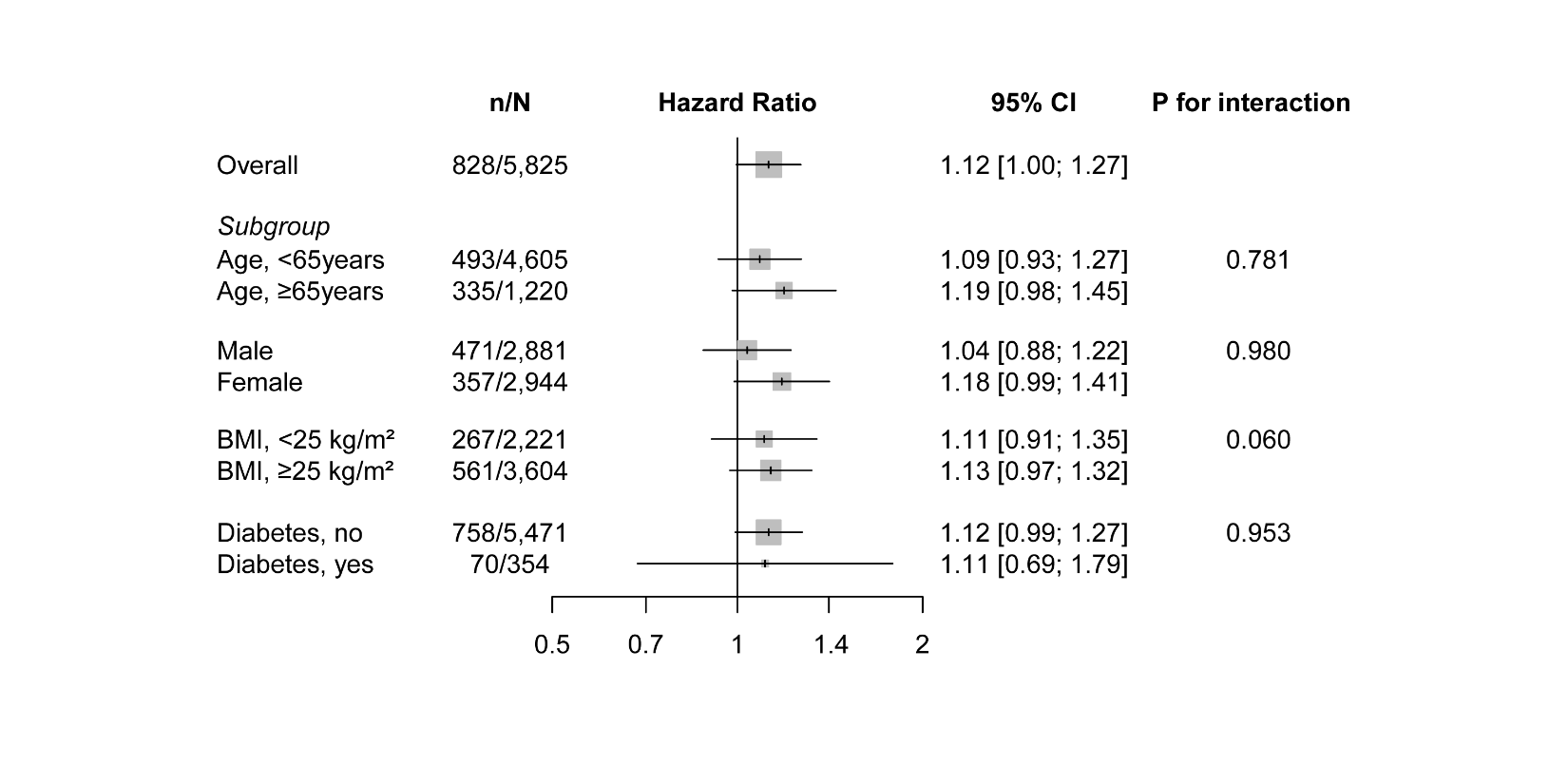


HRs and 95% CIs were derived from Cox proportional hazards regression models.

Hazard ratios were adjusted for age, sex, BMI, alcohol, eGFR, type 2 diabetes, fasting glucose, C-Peptide, triglycerides, HDL cholesterol, and hs-CRP.

Abbreviations: BMI, body mass index; hs-CRP, high sensitivity C-reactive protein; eGFR, estimated glomerular filtration rate; HDL, high-density lipoprotein.

**Methods**

**Study design and population**

For this study, we analyzed data from the Prevention of Renal and Vascular End-stage Disease (PREVEND) study, a prospective, population-based cohort of Dutch men and women aged 28 to 75 years.^1^ In total, 8,592 subjects were screened at the first examination (1997-1998) and each 3 to 4 years thereafter. Of these, 6,894 subjects returned for the second examination (2001-2003), which was considered as the baseline for the present study. We excluded those with non-fasting laboratory measurements, with missing values of KBs, and with KB levels of <2.5^th^ and >97.5^th^ percentile,^2^ leaving 6,079 participants. The PREVEND study conformed to the principles drafted in the Helsinki declaration. Local medical ethics committee approval was obtained (approval number: MEC96/01/022), and all participants provided informed consent.

**Data collection**

The procedures at each examination in the PREVEND study have been described in detail previously.^3^ In brief, each examination included 2 visits to an outpatient clinic separated by 3 weeks. Before the first visit, all participants completed a questionnaire regarding demographic characteristics, smoking habits and alcohol consumption. During the first visit, participants' height and weight were assessed. During each visit, blood pressure was measured and the average value was used.

**Assessment of covariates**

Body mass index (BMI) was calculated as the ratio of weight to height squared (kg/m^2^). Smoking status was defined as self-reported never, former or current smoking. Alcohol consumption was evaluated as alcoholic drinks per day, with one drink being considered to be equivalent to 10 g of alcohol regardless of the type of beverage,^4^ and was categorized in (i) no/rarely; (ii) 0.1–10 g per day (occasional to light); (iii) 10–30 g per day (moderate), and (IV) >30 g per day (heavy).^5^ Type 2 diabetes was defined as a fasting plasma glucose level of ≥7.0 mmol/L, non-fasting glucose ≥11.1 mmol/L, or the use of glucose-lowering drugs.^6^

Glucose was determined using dry chemistry (Eastman Kodak, Rochester, NY).^7^ C-peptide was measured in plasma with an electrochemiluminescent immunoassay, using a Cobas e602 (Roche Modular E, Roche Diagnostics, Mannheim, Germany).^8^ Triglycerides were measured enzymatically (Abbott Laboratories, Abbott Park, IL, United States) and high-density lipoprotein (HDL) cholesterol was measured utilizing a homogenous method (direct HDL; Aeroset System; Abbott Laboratories, Abbott).^9^ High sensitivity C-reactive protein (hsCRP) was assessed utilizing nephelometry (Dade Behring Diagnostic, Marburg, Germany).^2^ Serum creatinine was measured by an isotope dilution mass spectrometry traceable enzymatic method on a Roche Modular analyzer using reagents and calibrators from Roche (Roche Diagnostics, Mannheim, Germany). Serum cystatin C was measured by the Gentian Cystatin C Immunoassay (Gentian AS, Moss, Norway) on a Modular analyzer (Roche Diagnostics).^10^ Estimated glomerular filtration rate (eGFR) was calculated with the 2012 combined creatinine cystatin C-based Chronic Kidney Disease Epidemiology (CKD-EPI) Collaboration equation, taking into account age, sex and race.^11^

**Assessment of ketone bodies**

Total KBs (AcAc, β-OHB, acetone) were determined by NMR spectroscopy at Labcorp (Morrisville, NC). NMR spectra were collected on Vantera Clinical Analyzers,^12^ and concentrations were calculated using an optimized NMR LipoProfile test version (LP4 algorithm) as described previously.^13^

**Ascertainment of cancer outcomes**

The primary outcome was the incidence of overall cancer. Non-melanoma skin cancer was excluded from the definition of overall cancer due to its high prevalence and benign prognosis when compared to other malignancies.^14^ Secondary outcomes were the incidence of the most common site-specific cancers in our study population during the total follow-up (i.e., urinary tract and lung cancer). The incidence of colorectal cancer was additionally analyzed as a secondary outcome because the association between dietary ketosis and colorectal cancer was reported in previous studies.^15^ Subjects with a cancer diagnosis before baseline were excluded from the analyses of the corresponding cancer type and for overall cancer incidence. Data on cancer incidence were retrieved via linkage to Palga, the Dutch nationwide pathology databank. Palga data were obtained from the period 1971-2015.^16^ In addition to Palga data, we used the data of a self-reported questionnaire to exclude subjects with cancer before baseline. In case of multiple cancer diagnoses during follow-up, the earliest cancer diagnosis after baseline was used to index overall cancer. Subjects were censored at the end of follow-up (December 31, 2015) or at the date of non-cancer death, whichever occurred first.

**Statistical analyses**

Baseline characteristics are shown according to tertiles of KB concentrations. Continuous data are presented as mean with SD or as median with IQR in case of skewed distribution. Categorical data are presented as percentages. Trend analysis of baseline characteristics within KB strata was performed by linear regression or Kruskal-Wallis test for continuous variables and linear-by-linear association χ2 test for categorical variables.

KB concentrations were analyzed as a continuous term per 1-unit increment of the log2-transformed KB (i.e. per doubling of KB). The missingness of the covariates was limited (Table S1). We imputed the missing values of the categorical covariates by adding a category of missing, and we used listwise deletion to handle the missingness of the continuous covariates in regression analyses. We first selected potential confounders based on previous literature and the significant results of univariable linear regression and Cox regression analyses (Table S2). We then continued to perform multivariable Cox models with further adjustments for confounders associated with both KB and cancer outcomes in our data. In model 1, we calculated crude hazard ratios (HRs). In model 2, we calculated age- and sex- adjusted HRs. In model 3, we additionally adjusted for coexisting conditions including BMI, alcohol consumption, eGFR, and type 2 diabetes. Models 4a and 4b were expanded from model 3 by including additional adjustments of metabolic profiles (i.e., fasting glucose, C-Peptide, triglycerides and HDL cholesterol) and inflammatory activity characterized by hs-CRP, respectively. Several sensitivity analyses were conducted. First, because AcAc and acetone can be less stable during long-term storage at -70°C,^13^ we specifically examined β-OHB as the study exposure and remodeled the associations. Second, we explored possible effect modification by clinically important covariates on the associations of KBs and overall cancer incidence by fitting Cox models containing both main effects and the cross-product terms with KBs in the model adjusted for all confounders. Third, given the possible bidirectional link between KBs and inflammation,^17,18^ we considered three scenarios of hs-CRP, a marker portraying acute inflammation phase, (1) as a confounder, (2) as a mediator, and (3) as both (Figure S1). To further investigate the potential mediating role of hs-CRP, we conducted a mediation analysis using the *mediation* R package within the counterfactual framework described by Imai *et al*.^19^

All P-values are two-tailed. A P-value of <0.05 is considered statistically significant, except in the univariable analyses where we applied a threshold for P-value of <0.10 to identify candidate variables for subsequent multivariable analyses. All analyses were conducted using the statistical package IBM SPSS (v.22; SPSS, Chicago, IL, USA), and R (v.4.3.1, Boston, MA, USA).

**References**

1. Lambers Heerspink HJ, Brantsma AH, De Zeeuw D, Bakker SJL, De Jong PE, Gansevoort RT. Albuminuria assessed from first-morning-void urine samples versus 24-hour urine collections as a predictor of cardiovascular morbidity and mortality. *Am J Epidemiol*. 2008;168(8):897-905.

2. Szili-Torok T, de Borst MH, Garcia E, et al. Fasting Ketone Bodies and Incident Type 2 Diabetes in the General Population. *Diabetes*. 2023;72(9):1187-1192.

3. Hillege HL, Janssen WMT, Bak AAA, et al. Microalbuminuria is common, also in a nondiabetic, nonhypertensive population, and an independent indicator of cardiovascular risk factors and cardiovascular morbidity. *J Intern Med*. 2001;249(6):519-526.

4. Gruppen EG, Bakker SJL, James RW, Dullaart RPF. Serum paraoxonase-1 activity is associated with light to moderate alcohol consumption: The PREVEND cohort study. *Am J Clin Nutr*. 2018;108(6):1283-1290.

5. Koning SH, Gansevoort RT, Mukamal KJ, Rimm EB, Bakker SJL, Joosten MM. Alcohol consumption is inversely associated with the risk of developing chronic kidney disease. *Kidney Int.* 2015;87(5):1009-1016.

6. Expert Committee on the Diagnosis and Classification of Diabetes Mellitus. Report of the expert committee on the diagnosis and classification of diabetes mellitus. *Diabetes Care*. 2003;26:s5-s20.

7. Groothof D, Post A, Polinder-Bos HA, et al. Muscle mass and estimates of renal function: a longitudinal cohort study. *J Cachexia Sarcopenia Muscle*. 2022;13(4):2031-2043.

8. Sokooti S, Kieneker LM, de Borst MH, et al. Plasma c-peptide and risk of developing type 2 diabetes in the general population. *J Clin Med*. 2020;9(9):1-13.

9. Szili-Torok T, Bakker SJL, Tietge UJF. Normal fasting triglyceride levels and incident type 2 diabetes in the general population. *Cardiovasc Diabetol*. 2022;21(1).

10. Kieneker LM, Bakker SJL, de Boer RA, Navis GJ, Gansevoort RT, Joosten MM. Low potassium excretion but not high sodium excretion is associated with increased risk of developing chronic kidney disease. *Kidney Int.* 2016;90(4):888-896.

11. Inker LA, Schmid CH, Tighiouart H, et al. Estimating Glomerular Filtration Rate from Serum Creatinine and Cystatin C. *N Engl J Med*. 2012;367(1):20-29.

12. Matyus SP, Braun PJ, Wolak-Dinsmore J, et al. NMR measurement of LDL particle number using the Vantera Clinical Analyzer. *Clin Biochem*. 2014;47(16-17):203-210.

13. Garcia E, Shalaurova I, Matyus SP, et al. Ketone bodies are mildly elevated in subjects with type 2 diabetes mellitus and are inversely associated with insulin resistance as measured by the lipoprotein insulin resistance index. *J Clin Med*. 2020;9(2).

14. Apalla Z, Nashan D, Weller RB, Castellsagué X. Skin Cancer: Epidemiology, Disease Burden, Pathophysiology, Diagnosis, and Therapeutic Approaches. *Dermatol Ther (Heidelb).* 2017;7:5-19.

15. Dmitrieva-Posocco O, Wong AC, Lundgren P, et al. β-Hydroxybutyrate suppresses colorectal cancer. *Nature*. 2022;605(7908):160-165.

16. Casparie M, Tiebosch ATMG, Burger G, et al. Pathology databanking and biobanking in The Netherlands, a central role for PALGA, the nationwide histopathology and cytopathology data network and archive. *Cell Oncol.* 2007;29(1):19-24.

17. Kolb H, Kempf K, Röhling M, Lenzen-Schulte M, Schloot NC, Martin S. Ketone bodies: from enemy to friend and guardian angel. *BMC Med*. 2021;19(1).

18. Puchalska P, Crawford PA. Multi-dimensional Roles of Ketone Bodies in Fuel Metabolism, Signaling, and Therapeutics. *Cell Metab*. 2017;25(2):262-284.

19. Imai K, Keele L, Tingley D. A General Approach to Causal Mediation Analysis. *Psychol Methods*. 2010;15(4):309-334.
